# A review on the novel biomarkers of systemic lupus erythematosus discovered via metabolomic profiling

**DOI:** 10.3389/fimmu.2024.1443440

**Published:** 2024-11-06

**Authors:** Yinghong Liu, Xiaojuan Yang

**Affiliations:** ^1^ Department of Rheumatology, Chongqing University Central Hospital, Chongqing, China; ^2^ Department of Rheumatology, Chongqing Emergency Medical Center, Chongqing, China

**Keywords:** systemic lupus erythematosus, metabolomics, pathogenesis, lipid metabolism, amino acid metabolism

## Abstract

Systemic lupus erythematosus (SLE) is a multifaceted autoimmune disease affecting various body organs and systems. The diagnosis of SLE and its complications is based on evident clinical symptoms, serological marker levels, and pathological findings. Some serological markers have a low sensitivity and specificity, and biopsy procedures are invasive in nature. Hence, metabolomics has emerged as a valuable tool for SLE screening and categorization. Its application has contributed significantly to identifying SLE pathogenesis, improving clinical diagnosis, and developing treatment approaches. This review provides an overview of the utilization of metabolomics in the study of SLE, focusing on advancements in understanding the disease’s pathogenesis, aiding in diagnosis, and monitoring treatment efficacy.

## 1 Introduction

Systemic lupus erythematosus (SLE) is an autoimmune disease with a complex pathogenesis, and it has a low clinical cure rate, involving multiple body organs and systems (1). It is characterized by clinical manifestations such as long-term low-grade fever or recurring high fever, butterfly-shaped erythema on the face, oral ulcers, kidney lesions, and arthritis (2). Challenges persist in diagnosing and managing SLE due to its high heterogeneity (3). The global incidence rate of SLE ranges from 1.5 to 11.0 per 100,000 individuals per year. It is more prevalent in young women, with a male-to-female ratio of approximately 1:9 (4). Despite extensive research over the years, the exact cause of SLE remains unclear, highlighting the urgent need for highly sensitive and specific diagnostic biomarkers (5). Metabolomics, a field utilizing advanced scientific techniques like mass spectrometry, aims to quantitatively or qualitatively analyze the metabolites present in biological systems (6). This approach allows for the assessment of dynamic bodily functions and metabolic indicators. The research methodology typically involves sample collection, metabolite identification, and data acquisition and analysis (7). Metabolomics research specimens typically include serum, feces, urine, etc., which have the advantages of easy acquisition and low cost. As a result, the metabolomics has seen rapid development in recent years (8). The research aimed to explore the advancements in metabolomics studies on SLE with a focus on identifying disease-specific metabolic alterations, understanding their role in SLE pathophysiology, and assessing the potential of metabolomics in enhancing diagnostic and therapeutic strategies.

## 2 Metabolomics

Metabolomics is a systematic biological method that utilizes omics analytical techniques to identify specific metabolites or metabolomes in primary research specimens such as blood, urine, and feces (9). Metabolomics complements data from genomics, transcriptomics, and proteomics, and it is widely used in various diseases (10). For detecting and analyzing endogenous metabolites in biological fluids, the main analytical methods in metabolomics include nuclear magnetic resonance (NMR) spectroscopy, which encompasses hydrogen, carbon, and phosphorus spectra, gas chromatography-mass spectrometry (GC-MS), liquid chromatography-mass spectrometry (LC-MS), and capillary electrophoresis-mass spectrometry (CE-MS) (11). NMR is a non-destructive analytical technique that offers both structural information and quantitative data on metabolites. Despite its relatively low sensitivity, NMR holds significant value in metabolomics research, particularly in the metabolic network analysis of complex biological systems, owing to its high reproducibility and the lack of complex sample preprocessing requirements (12). GC-MS is an effective method for analyzing volatile and semi-volatile metabolites. By integrating the separation capabilities of gas chromatography with the detection capabilities of mass spectrometry, GC-MS provides high-resolution separation and precise identification of metabolites. This technique is particularly well-suited for the analysis of small molecules, including fatty acids and amino acids. LC-MS is among the most widely employed techniques in metabolomics, noted for its high sensitivity and resolution. This method is effective for detecting both polar and non-polar small molecule metabolites and is extensively utilized for the qualitative and quantitative analysis of metabolites in biological samples. CE-MS offers high resolution and selectivity, which facilitates the effective separation of metabolites in complex samples—a critical aspect of metabolomics research. CE-MS is particularly proficient in the analysis of amino acids, nucleotides, and their derivatives. Additionally, it offers advantages such as reduced sample consumption and rapid analysis speed, establishing it as an important complementary technology in the field of metabolomics (13–15). In addition to the primary tools mentioned above, emerging technologies such as Mass Spectrometry Imaging (MSI) and High-Resolution Mass Spectrometry (HRMS) demonstrate significant potential in metabolomics research (16). These techniques can offer more detailed and accurate information regarding the distribution and dynamic changes of metabolites. The advancements in MS and NMR technologies, coupled with the development of sophisticated bioinformatics tools for data analysis, have significantly bolstered the capabilities of metabolomics in identifying metabolic changes linked to systemic diseases (17). These technologies can help researchers construct detailed metabolic profiles that can shed light on disease mechanisms, aid in the discovery of novel biomarkers, and offer novel therapeutic targets.

## 3 Application of metabolomics in SLE

### 3.1 Application of metabolomics in the pathogenesis of SLE

The pathogenesis of SLE has not been completely elucidated. Furthermore, it is associated with multiple factors including genetic susceptibility within families, environmental changes, immune influences, and estrogen levels (5, 18, 19). Metabolomics offers unparalleled insights on the metabolic dysregulations in SLE (20). Metabolomics can elucidate the metabolic characteristics and biomarkers associated with SLE, thereby offering new opportunities for early diagnosis and disease monitoring. This non-invasive methodology not only alleviates the burden on patients but also improves the accuracy and reliability of biomarkers, thereby establishing a foundation for the development of personalized treatment plans. Metabolomics contributes to understanding SLE pathogenesis via various pathways including lipid metabolism, amino acid metabolism, and gut microbiome metabolism.

Lipidomics, as a unique branch of metabolomics, can be used to identify spatiotemporal changes in lipid profiles and reveal complex causes at the molecular level (21–23). Lipid metabolism is the process of synthesis, digestion, degradation, and absorption of lipids (such as cholesterol, triglycerides, and fatty acids) and is essential for almost every cellular process (24). Therefore, an imbalance of lipid homeostasis can lead to SLE. Lipid metabolism alterations play a role in SLE development and progression. Recently, LC-MS/MS analysis has been utilized, and significant differences in lipidomic profiles were observed between patients with SLE and healthy controls (HCs). Furthermore, individuals with SLE present with significantly high levels of sphingolipids, including ceramides, ceramide phosphoinositols, and most diradylglycerol classes (25). By contrast, patients with SLE exhibited higher apolipoprotein B, apolipoprotein C, apolipoprotein D, apolipoprotein E, and apolipoprotein L1 levels than HCs (25). Sphingolipids and apolipoproteins play an irreplaceable role in atherosclerosis (26, 27). Based on the lipid metabolism imbalance observed in patients with atherosclerosis, the hyperlipidemia environment caused by abnormal lipid metabolism may be related to SLE pathogenesis (28). Therefore, disruptions in lipid metabolism, particularly within sphingolipid metabolism, along with altered apolipoprotein levels, play a role in driving the disease activity associated with SLE (25). Research has demonstrated the significant role of lipids and lipid metabolites in the immune system, particularly in indirectly regulating antigen presentation and cytokines that act on immune cells (29). Dysfunctional immune cells, such as T cells and B cells, contribute to abnormal immunity toward inflammatory mediators and loss of tolerance to self-antigens, which are key factors in the pathogenesis of SLE (30, 31). Studies have indicated that lipidomic analysis of peripheral blood mononuclear cells (PBMC) from SLE patients revealed altered lipid metabolism, including a notable increase in lysophospholipids, a decrease in plasmalogens, and variations in phosphatidylserine levels compared to those in healthy controls (32). Abnormal amino acid metabolism is an important mechanism in SLE pathogenesis. In the early or middle-to-late stages of SLE, abnormalities in amino acid metabolism occur, leading to elevated levels of amino acids that cause immune dysfunction. Iwasaki et al. (33) conducted a study on patients with SLE and rheumatoid arthritis and a healthy population. They analyzed the metabolomic profiles of plasma samples using LC-MS and capillary electrophoresis-mass spectrometry. Results showed that plasma histidine levels were negatively associated with the American College of Rheumatology damage index. Patients with SLE who had high levels of anti-double-stranded DNA antibodies or concurrent lupus nephritis (LN) had significantly lower plasma histidine levels than those with low levels of anti-double-stranded DNA antibodies or those without LN. The solute carrier family 15, member 4 (SLC15A4) gene is involved in histidine transport and is one of the SLE susceptibility genes (34). Histidine concentration in lysosomes regulates TLR7-induced B-cell production of type I IFN, thereby supporting its homeostatic role in SLE pathogenesis (35). Low histidine levels may be associated with the disease mechanism of SLE. Furthermore, environmental factors play an important role in initiating and promoting SLE-associated autoimmune reactions (36). The gut microbiota affects host immune homeostasis by altering metabolites, and changes in metabolites typically affect local or systemic immune responses (37). The microbiota of patients with SLE is disrupted, with a significant decrease in the ratio of Firmicutes-to-Bacteroidetes (38). Patients with SLE exhibit dysbiosis of the gut microbiota and an imbalance in intestinal ecology, with an increased relative abundance of pathogenic bacteria (such as *Lactobacillus reuteri*) and a decrease in bacteria with anti-inflammatory effects (such as Lactobacilli) (39). Unlike other Lactobacilli, *L. reuteri* can exacerbate autoimmune manifestations by participating in the type I interferon pathway (40). Mu et al. (41) performed fecal metabolomics study of MRL/lpr mice. Results showed a significant reduction in the number of lactobacilli in MRL/lpr mice. By increasing lactobacilli, it was possible to correct intestinal leakiness, enhance interleukin-10 secretion, improve renal function, and extend the survival time of the mice.

### 3.2 Auxiliary role of metabolomics in the diagnosis of SLE

Conventional serological markers used for diagnosing and monitoring SLE, such as antinuclear antibodies, anti-double-stranded DNA antibodies, and complement levels, have limited sensitivity and specificity. Renal biopsy remains the gold standard for determining the prognosis of patients with kidney involvement (42). However, since it is an invasive procedure, it carries a certain risk of harm. Therefore, novel biomarkers for the diagnosis, classification, and prognostic identification of SLE should be explored.

#### 3.2.1 Lipidomics

Lipidomics plays an important role in identifying the biomarkers of SLE. Lipids are the most significantly altered category of serum metabolites in patients with SLE (43). A previous study utilizing high-performance liquid chromatography-tandem mass spectrometry discovered that serum ceramides and trimethylamine N-oxide (TMAO) levels were significantly higher in patients with active SLE (44). Ceramides, as second messengers based on sphingolipids, are associated with oxidative stress, which is 172 involved in apoptosis signaling pathways (45). Elevated serum ceramide levels may facilitate cell apoptosis, thereby exacerbating SLE progression. Abnormal lipid metabolism is associated with an increased risk of atherosclerosis in patients with SLE. Thus, SLE is often associated with hyperlipidemia (46). TMAO is a product of trimethylamine oxidation, a common metabolic product derived from choline metabolism by the gut microbiota. Circulating TMAO levels are closely associated with atherosclerosis, potentially by increasing cholesterol accumulation within macrophages, leading to atherosclerosis (47). Ceramides and TMAO can be therapeutic targets in SLE. Zhang et al. (48) identified 28 characteristic metabolites that differentiate SLE from LN using ultra-high performance liquid chromatography-tandem mass spectrometry methods. Five of these serum metabolites had a high auxiliary discrimination performance, providing more references for LN auxiliary diagnosis and offering additional support for pathophysiological research on the progression of SLE to LN. The study conducted metabolomic and lipidomic evaluations using LC-MS/MS on 133 patients with SLE and 30 HCs. In total, 13 differential metabolites were identified as potential biomarkers of SLE. The area under the receiver operating characteristic curve of lysophosphatidyl ethanolamine for diagnosing SLE as 0.903 (49). Previous research has shown that lysophosphatidyl ethanolamine can be used to identify patients with SLE and has auxiliary diagnostic value for SLE (50). In addition, Study analyzed the lipid profiles of 71 young female patients with SLE and found significantly increased levels of triglycerides and very low-density lipoprotein cholesterol (VLDL-C). In contrast, total cholesterol, high-density lipoprotein cholesterol (HDL-C), low-density lipoprotein cholesterol (LDL-C), and apolipoproteins A and B were significantly decreased. Furthermore, SLE disease activity was significantly correlated with these lipid indicators (51). Consequently, patients with SLE should regularly monitor their blood lipid levels, and in cases of hyperlipidemia, prompt intervention is necessary.

#### 3.2.2 Amino acid metabolism

**Figure 1:**
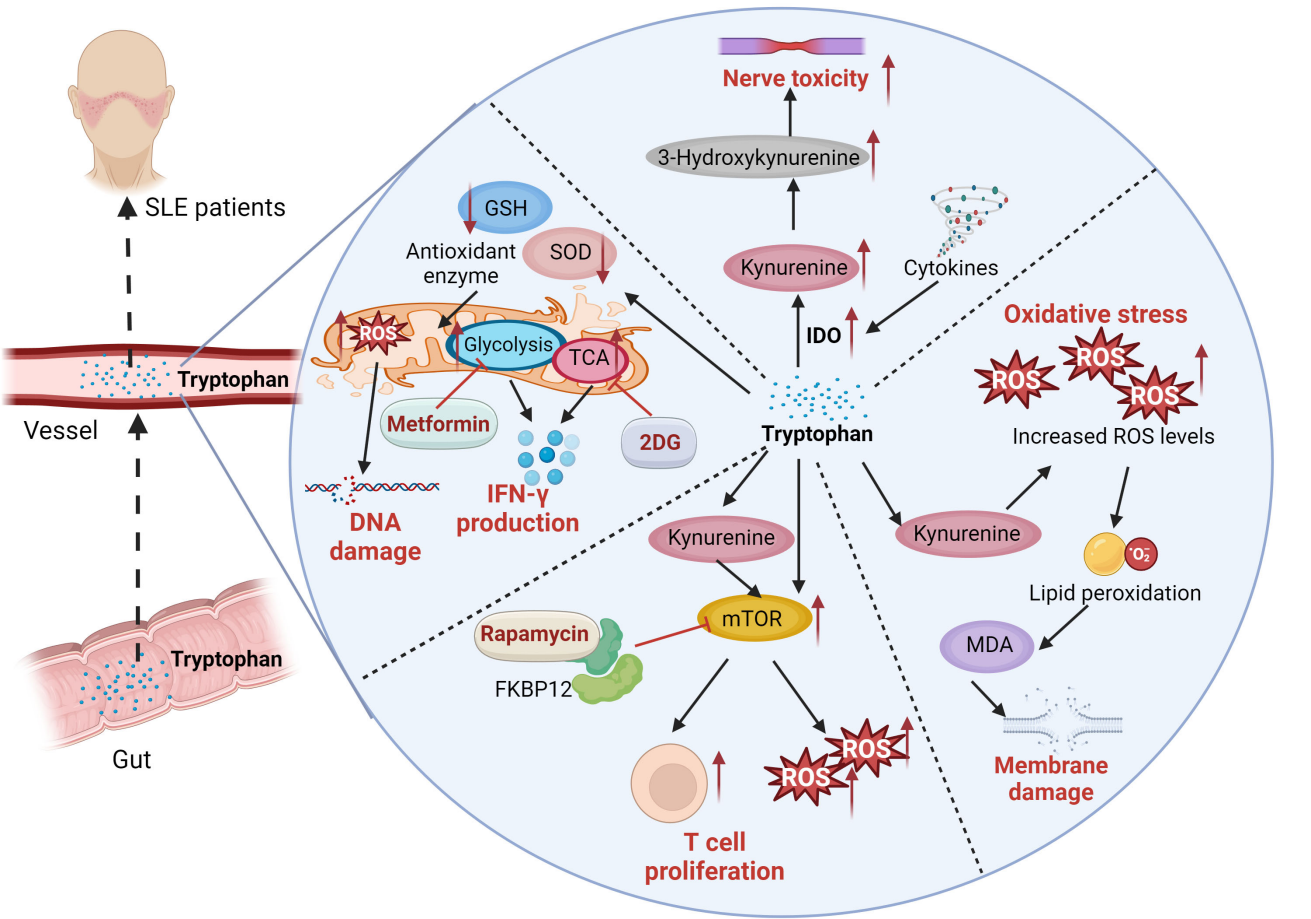
The serum levels of kynurenine, kynurenic acid, 3-hydroxyanthranilic acid, 3-hydroxykynurenine, were significantly elevated in SLE patients. Tryptophan is an essential amino acid primarily metabolized via the kynurenine pathway. Tryptophan is primarily metabolized via the indoleamine 2,3-dioxygenase (IDO) pathway into kynurenine, which can be further converted into 3-hydroxykynurenine and quinolinic acid. These neurotoxic and pro-oxidative metabolites promote the production of reactive oxygen species (ROS), leading to cellular oxidative stress. ROS induce lipid peroxidation, leading to the formation of malondialdehyde (MDA), which signifies damage to the cell membrane. SLE Patients exhibit intracellular glutathione depletion and activation of downstream metabolic sensors due to oxidative stress. Rapamycin, also known by its generic name sirolimus, inhibits antigen-induced T-cell proliferation. Rapamycin forms a high-affinity complex with its cellular receptor FKBP12, which is overexpressed in lupus T cells, thereby blocking mTOR activation to improved disease activity in SLE patients. CD4(+) T cells play a crucial role in the progression of SLE. Both metformin, a mitochondrial metabolism inhibitor, and 2-deoxy-D-glucose (2DG), a glucose metabolism inhibitor, have been shown to reduce the production of interferon-γ (IFN-γ). When used in combination, these agents normalize T cell metabolism and reverse disease biomarkers.

The differences in amino acid metabolism between SLE patients and healthy individuals highlight disease-related metabolic disturbances, which may offer new insights into the pathophysiological mechanisms underlying SLE. These variations in amino acid metabolism establish a basis for future investigations into potential diagnostic biomarkers and risk assessment strategies for SLE (52). Li et al. (53) discovered that patients with SLE exhibit higher serum taurine levels compared to healthy controls. This elevated taurine level is positively correlated with disease activity. Taurine plays a role in promoting the expression of type I IFN-induced genes, activating lymphocytes, and enhancing the secretion of autoantibodies, which can result in proteinuria and severe nephritis. The main mechanism involves the augmentation of type I IFN production mediated by plasmacytoid dendritic cells, thus contributing to the pathogenesis of SLE. A specific serum taurine value can be utilized for diagnosing SLE and assessing disease activity (54). Tryptophan is an essential amino acid primarily metabolized via the kynurenine pathway. Its metabolites are crucial in regulating immune responses, managing inflammation, and mitigating oxidative stress. Studies have demonstrated that tryptophan metabolism is altered in patients with SLE. Compared to controls, serum tryptophan levels are significantly reduced, while kynurenine concentrations are elevated. The degree of tryptophan catabolism correlates with neopterin levels or the disease activity index. Furthermore, tryptophan depletion may be linked to neurological and psychiatric disorders in patients with SLE (55). Tryptophan, an essential amino acid, is primarily metabolized via the indoleamine 2,3-dioxygenase (IDO) pathway into kynurenine, which can be further converted into 3-hydroxykynurenine and quinolinic acid. These neurotoxic and pro-oxidative metabolites promote the production of reactive oxygen species (ROS), leading to cellular oxidative stress (56). Eryavuz Onmaz D et al. (57) investigated the metabolite levels of the kynurenine pathway in patients with SLE and found that serum levels of knurenine, kynurenic acid, 3-hydroxyanthranilic acid, 3-hydroxykynurenine, and quinolinic acid were significantly elevated in SLE patients compared to healthy controls. These findings suggest that the imbalance of kynurenine pathway metabolites may be closely associated with the pathogenesis and clinical manifestations of SLE. SLE patients exhibit intracellular glutathione depletion and activation of downstream metabolic sensors due to oxidative stress (58). N-acetylcysteine (NAC) reverses glutathione depletion and has therapeutic effects in SLE, with kynurenine identified as a significant predictor of NAC efficacy (59). Rapamycin, also known by its generic name sirolimus, inhibits antigen-induced T-cell proliferation and has been developed as a medication for the prevention of organ transplant rejection. Rapamycin forms a high-affinity complex with its cellular receptor FKBP12, which is overexpressed in lupus T cells, thereby blocking mTOR activation to improved disease activity in SLE patients (60, 61). CD4(+) T cells play a crucial role in the progression of SLE (62). Both metformin, a mitochondrial metabolism inhibitor, and 2-deoxy-D-glucose (2DG), a glucose metabolism inhibitor, have been shown to reduce the production of interferon-γ (IFN-γ). When used in combination, these agents normalize T cell metabolism and reverse disease biomarkers (63). The detailed mechanisms of tryptophan in SLE disease was illustrated in **Figure 1**. And experiments with lupus-prone mouse models have shown that abnormalities in tryptophan metabolism can activate autoimmune diseases. Supplementing tryptophan in lupus-prone mice alters the gut microbiota and upregulates the expression of genes related to intestinal epithelial integrity (54). These findings indicate a potential link between gut microbiome dysbiosis and autoimmune activation. Exogenous tryptophan supplementation has the potential to serve as a novel adjuvant treatment for SLE. By modulating tryptophan metabolic pathways, it may enhance patients’ clinical symptoms and overall quality of life. Nevertheless, further research is required to confirm its safety and efficacy prior to clinical implementation. In addition, study employed ultra-performance liquid chromatography-tandem mass spectrometry (UPLC-MS/MS) to analyze amino acid levels in plasma, revealing significant changes in the plasma amino acid profile of LN patients. The combined model incorporating histidine, lysine, and tryptophan, as well as the model consisting of arginine (Arg), valine (Val), and tryptophan (Trp), both demonstrated excellent diagnostic performance based on alterations in plasma amino acid levels. This approach offers a novel perspective for the early identification, prevention, treatment, and management of LN (64).

#### 3.2.3 Urinary metabolism

Urine sample collection has several advantages. For example, this method requires a minimal sample volume, its sample is easy to collect, and it is noninvasive and cost-effective. Urinary metabolomics allows for a convenient and efficient analysis of endogenous metabolites in patients with SLE, aiding in the identification of physiological variations during disease onset, active phases, or treatment. Urinary metabolites can serve as biomarkers for clinical diagnosis and activity assessment (65). Kalantari and colleagues employed 1H-nuclear magnetic resonance methodology to analyze urinary metabolic variations in patients with LN, SLE, and HCs. They observed a significant decrease in urinary β-alanine excretion in LN patients compared to those with SLE and HCs, suggesting a potential association between diminished renal carnosine levels and LN. Carnosine, synthesized in the kidneys, plays a crucial role in maintaining kidney function. Lower carnosine levels may render the kidneys more vulnerable to oxidative stress-induced damage (66). Liu et al. (67) examined 15 samples obtained from MRL/lpr mice of varying ages. The results indicated elevated levels of urea and urate in the mice at 13 weeks. Elevated urea levels have been linked to impaired kidney function, while urates can trigger inflammatory pathways and the deposition of autoantibodies in the kidneys, leading to decreased glomerular filtration rates (68). A significant portion of individuals with SLE develop renal damage, characterized by indicators such as glomerular and tubulointerstitial lesions (69). High levels of uric acid and urates serve as valuable indicators for evaluating renal function in SLE patients. Rojo-Sánchez et al. (70). employed a non-targeted metabolomics approach to analyze urine samples from various groups, including SLE patients without renal impairment, LN patients, and healthy controls. They observed significant differences in multiple metabolic pathways between LN patients and SLE patients without renal impairment, notably in primary bile acid biosynthesis and branched-chain amino acid synthesis and degradation. Furthermore, metabolites such as palmitic acid, glycolic acid, and glutamic acid were found to effectively distinguish SLE patients without renal impairment from LN patients.

#### 3.2.4 Gut microbiota metabolism

Research on the metabolic changes within the gut microbiome enhances our understanding of the pathophysiological mechanisms underlying SLE and offers potential insights for the diagnosis and treatment of this disease (71). Yuan et al. (72) used four analytical platform methods, including 2DLC-MS and GC×GC-MS, to compare fecal metabolites among castrated (androgen-depleted) male, intact (androgen-supplemented) male, and female mice. They found that compared with castrated androgen-depleted male mice, female mice exhibited higher mortality rates. Furthermore, the two groups have significantly lower survival rates than intact male mice. This protective effect of androgens may be associated with microbiota metabolite changes. Androgens influence the gut microbiome, which reciprocally affects them. Histidine metabolism is one of the distinguishing factors between female and castrated androgen-depleted male mice, compared with intact male mice. Histidine is a precursor to histamine, an effector molecule that can either mediate inflammation or modulate responses based on target cells and the types of histamine receptors expressed. Probiotics and endogenous bacterial species can produce histamine and influence gut immune responses (73). The assessment of histamine levels can provide reference value for diagnosing SLE. Yan et al. utilized GC-MS and identified significant differences in the fecal metabolome between patients with SLE and HCs (74).

### 3.3 Application of metabolomics in SLE treatment

In SLE treatment, Western medicine primarily uses immunosuppressants and steroids, which can lead to significant liver and kidney damage with prolonged usage (75). Traditional Chinese medicine is effective in slowing SLE progression and alleviating symptoms, predominantly relying on clinical experience without a systematic syndrome classification and treatment approach (76, 77). Currently, there is a burgeoning interest in pharmacometabolomics research concerning drugs used for SLE treatment. Treadwell EL et al. (78) conducted serum metabolite analysis on patients with SLE and healthy individuals using LC-MS/MS metabolomics methods, which can identify dehydroepiandrosterone sulfate as a differential metabolite. Dehydroepiandrosterone sulfate, a secretory product of dehydroepiandrosterone, is found at lower levels in patients with SLE (78). Short-term dehydroepiandrosterone supplementation has been found to be beneficial in treating mild to moderate SLE (79). Hence, DHEA can provide a novel avenue for SLE treatment. Saegusa et al. (80) used GC-MS to examine differences in serum metabolites between patients with SLE and HCs. Results showed variations in the levels of 25 metabolites, with significant differences observed in glutamic acid levels. Glutamic acid levels were significantly elevated in the splenic mononuclear cells of MRL/lpr mice and the peripheral blood mononuclear cells of patients with SLE. These phenomena lead to decreased intracellular glutamic acid levels and improvement in renal pathological lesions (81). Lowering glutamic acid levels can be a novel therapeutic approach for SLE.Wei et al. (82) conducted an analysis of urine samples from MRL/lpr mice modeling SLE, SLE mice treated with prednisolone acetate, SLE mice treated with traditional Chinese medicine, and control mice using GC-MS. They observed differences in urine metabolites between the SLE model group and the other groups. Anomalies in metabolic pathways were primarily evident in disrupted energy metabolism and amino acid metabolism. Disrupted energy metabolism can generate a large quantity of pro-inflammatory substances, thereby promoting SLE progression. Perturbed amino acid metabolism can result in disturbances in physiological and immune functions, which facilitates SLE progression. These findings offer experimental support for further investigating the mechanisms of traditional Chinese medicine in treating SLE. Liu et al. (83) discovered that the detoxification, hemostasis, and nourishing Yin formula (JP) has favorable effects in SLE treatment. After 8 weeks of JP treatment, the MRL/lpr mice presented with significantly reduced urinary protein levels, periglomerular inflammatory cell infiltration, basement membrane thickening, and interstitial fibrosis. Metabolomics and intelligent pathway analysis showed that the process of renal injury might be associated with abnormalities in the farnesoid X receptor (FXR) pathway in lupus mice. FXR can be an effective therapeutic target for LN and renal fibrosis. JP might mediate renal FXR expression activation, thereby exerting anti-inflammatory and anti-fibrotic effects for LN prevention and treatment.

## 4 Conclusion

Metabolomics can detect disease biomarkers at the metabolic level, thereby facilitating early disease diagnosis and identifying clinical treatment strategies. Current research on SLE metabolomics includes investigating SLE pathogenesis, identifying unique metabolites for an accurate SLE diagnosis, and developing therapeutics with minimal adverse effects (**Table 1**). Metabolomics has been widely utilized in diverse disciplines including nutrition, environmental science, and botany. However, research on SLE is limited, and there is a lack of consensus on standardized methodologies. Differential metabolites distinguishing patients with SLE from healthy individuals have been identified. Nevertheless, the specific metabolites significantly associated with SLE pathogenesis remain elusive. Moreover, the underlying pathways and mechanisms via which these differential metabolites operate remain poorly understood. This underscores the need to perform more studies to elucidate the associated mechanisms comprehensively. Importantly, understanding these metabolic changes may inform personalized treatment strategies for SLE. By identifying the specific metabolic pathways involved in individual patients, metabolomics can facilitate the development of targeted treatment options, thereby advancing the objectives of precision medicine. Future research should prioritize the integration of metabolomic findings into clinical practice to enhance outcomes for patients with SLE.

**Table 1:** Overview of metabolomic studies of SLE and LN.

Major differential metabolites	Sample type	Diseases	Analytical platform	Reference
Sphingolipids and apolipoproteins	Blood	SLE	LC-MS/MS	Huang X et al. (25)
Plasmalogens, lysophospholipids, phosphatidylserines	PBMC	SLE	Shotgun lipidomics	Hu C et al. (32)
Histidine	Plasma	SLE	LC-MS/CE-MS	Iwasaki Y et al. (33)
ceramide, TMAO, xanthine	Serum	SLE	LC-MS	Li Y et al. (44)
Glycerolphospholipid, sphingomyelins	Serum	SLE/LN	UPLC-MS/MS	Zhang Y et al. (48)
Lysophosphatidylethanolamine, Dehydroepiandrosterone sulfate, 3,4-Dihydroxymandelaldehyde	Serum	SLE	LC-MS/MS	Zhang W et al. (49)
Taurine	Serum	SLE	MS	Li J et al. (53)
3-hydroxykynurenine, quinolinic acid, kynurenine, kynurenic acid, 3-hydroxyanthranilic acid	Serum	SLE	MS	Eryavuz Onmaz D et al. (56)
histidine, lysine, and tryptophan, arginine, valine, and tryptophan	Plasma	SLE/LN	UPLC-MS/MS	Guo ZS et al. (57)
Nicotinate, nicotinamide, beta-alanine	Urine	SLE/LN	NMR	Kalantari S et al. (59)
Urea, urate, and indole-3-lactate	Serum	MRL/lpr mice	GC/MS	Liu J et al. (60)
Glycolic acid, glutamic acid, Monopalmitin,	Urine	SLE/LN	LC-MS/GC-MS	Rojo-Sánchez A et al. (63)
Tryptophan, 5-hydroxytryptophan, serotonin	Feces	BWF1 mice	LC-MS/GC-MS	Yuan F et al. (64)
deoxycholic acid, erucamide, L-tryptophan putrescine	Feces	SLE	GC-MS	Yan R et al. (66)
Dehydroepiandrosterone sulfate	Serum	SLE	LC-MS/MS	Treadwell EL et al. (70)
Glutamic acid, urea, tyrosine, phosphate, glycerol	Serum	SLE	GC/MS	Saegusa J et al. (72)
Citric acid, malic acid, lactic acid, benzene, hippuric acid	Urine	MRL/lpr mice	GC-MS	Wei J et al. (74)
